# Neuromuscular electrical stimulation leads to physiological gains enhancing postural balance in the pre-frail elderly

**DOI:** 10.14814/phy2.12471

**Published:** 2015-07-30

**Authors:** Jean-Baptiste Mignardot, Thibault Deschamps, Camille G Le Goff, François-Xavier Roumier, Julien Duclay, Alain Martin, Marc Sixt, Michel Pousson, Christophe Cornu

**Affiliations:** 1 Laboratory up-Courtine, Swiss Federal Institute of Technology (EPFL)Lausanne, Switzerland; 2 Laboratory MIP (UPRES-EA4334), University of NantesNantes, France; 3 Laboratory CASP (INSERM-U1093), University of BurgundyBurgundy, France; 4 Laboratory PRISSMH, team LAPMA (EA 4561), University of Tolouse IIITolouse, France; 5 Geriatric Department, Hospital of Beaune (Burgundy)Beaune, France

**Keywords:** Muscle-tendon unit, neuromuscular electrical stimulation, postural balance

## Abstract

Physiological aging leads to a progressive weakening of muscles and tendons, thereby disturbing the ability to control postural balance and consequently increasing exposure to the risks of falls. Here, we introduce a simple and easy-to-use neuromuscular electrical stimulation (NMES) training paradigm designed to alleviate the postural control deficit in the elderly, the first hallmarks of which present as functional impairment. Nine pre-frail older women living in a long-term care facility performed 4 weeks of NMES training on their plantarflexor muscles, and seven nontrained, non-frail older women living at home participated in this study as controls. Participants were asked to perform maximal voluntary contractions (MVC) during isometric plantarflexion in a lying position. Musculo-tendinous (MT) stiffness was assessed before and after the NMES training by measuring the displacement of the MT junction and related tendon force during MVC. In a standing position, the limit of stability (LoS) performance was determined through the maximal forward displacement of the center of foot pressure, and related postural sway parameters were computed around the LoS time gap, a high force requiring task. The NMES training induced an increase in MVC, MT stiffness, and LoS. It significantly changed the dynamics of postural balance as a function of the tendon property changes. The study outcomes, together with a multivariate analysis of investigated variables, highlighted the benefits of NMES as a potential tool in combating neuromuscular weakening in the elderly. The presented training-based strategy is valuable in alleviating some of the adverse functional consequences of aging by directly acting on intrinsic biomechanical and muscular properties whose improvements are immediately transferable into a functional context.

## Introduction

Sarcopenia is a common aging process that leads to a set of neuromuscular alterations, which contribute to the reduction in functional capacities and autonomy levels (Hill 2001; Wroblewski et al. 2011; Rom et al. 2012). The recent definition of sarcopenia (Report of the European Working Group on Sarcopenia in Older People 2010) in relation to age suggests not only a decline in muscle mass (Rosenberg 1997) but also a decline in muscle functions (i.e., the force, the force control, and the force transmission) that disturb balance and motor control. The sarcopenia process is known to particularly affect muscles that are strongly involved in postural control, such as the knee and ankle extensor muscles (i.e., quadriceps and triceps surae muscles).

### Triceps surae and postural control

The triceps surae (TS) is of paramount importance for the control of standing and walking (Sutherland et al. 1980; Schieppati et al. 1994; Loram et al. 2004). Its activation leads to ankle extension (or plantarflexion [PF]) and therefore to the forward displacement of the center of plantar pressure (CoP) within the base of support (BoS) (Winter 1995; Loram et al. 2004). Consequently, it plays a central role in the adjustment of the anteroposterior (A-P) position of CoP, depending on the actual A-P position of the center of mass (CoM), in order to maintain the postural balance. In contrast, it can also generate a mismatch between the A-P position of CoP and CoM to create an imbalance or initiate movement, for example, during a body-reaching task or the transition from standing to walking (Winter 1995; Polcyn et al. 1998; Stapley et al. 2000).

In this context, the ability to fully and accurately mobilize CoP within BoS is conditioned by the torque produced by the muscles crossing the joint and their force transmission to the external environment through the tendon (Winter et al. 1998; Loram et al. 2004; Onambele et al. 2006; Melzer et al. 2009) from a strictly biomechanical point of view. Any alteration in these properties (force and transmission of force) could lead to adverse changes in postural balance in older adults (Onambele et al. 2006; Billot et al. 2010; Sarabon et al. 2013) and increase the risk of a fall.

### Postural control disorders with aging

Aging is associated with a decline in postural balance (Maki et al. 1994; Horak 2006) and in the control required for reaching, tilting, or goal-directed movements starting from a standing position (Darling et al. 1989; Paizis et al. 2008), which results in a greater likelihood of falls (Nachreiner et al. 2007; Robinovitch et al. 2013). Yet, the ability to amply move and finely control the position of CoP within the BoS is a prerequisite for performing daily movements, such as using a step or stool to reach higher places or stoop to pick up an object from the floor (Melzer et al. 2009). This capacity can be assessed by measuring the forward limit of stability (LoS), which is also reduced with aging (Cavanaugh et al. 1999; Kozak et al. 2003). Melzer et al. (2009) have shown that the reduction in the distance covered by CoP during a forward LoS task in older people is explained by a lack of force of the plantarflexor muscles (TS). This could explain, at least in part, falls that occur during dynamical reaching tasks. This point is consistent with the authors’ suggestion that the training of TS among older people should be considered in long-term-targeted fall-prevention programs.

### Neuromuscular damages with aging and activity-dependent plasticity

Physiological aging induces several musculo-tendinous (MT) damages, which can dramatically affect motor skills, including controlling postural balance (e.g., Lexell et al. 1983; Onambele et al. 2006; Lichtwark and Wilson 2008). The consequences of the overall damage can be summarized through three main outcomes: a reduction in the maximal force production (1), a decrease in fine control of force (2), and a lack of efficiency in force transmission from the muscle to the external environment through the tendon (3). The translation of these points is evidenced particularly by sarcopenia (Doherty 2003; Narici and Maffulli 2010) and by an increase in MT compliance (Lexell et al. 1983; Onambele et al. 2006; Narici et al. 2008). Several studies have demonstrated the exacerbated age-dependent MT damage in TS muscle (Simoneau et al. 2005; Onambele et al. 2006). These adverse effects can be slowed down by maintaining a sufficient level of physical activity (Hill 2001; Wroblewski et al. 2011) or through the physical therapy training protocols involving voluntary muscle contraction (Ferri et al. 2003; Simoneau et al. 2007). However, even if the activity-dependent muscle plasticity is still effective despite age (Hill 2001; Wroblewski et al. 2011), several constraints, which complicate the implementation of the muscle training protocol, must be considered to make it effective.

### Neuromuscular electrical stimulation in older adults

From a practical point of view, the physical training that targets the recovery of the muscle capacity requires the supervision of a qualified person and several appropriate devices that are space-consuming, expensive and, for most of them, joint-specific. From a clinical point of view, the use of these devices also requires dynamic muscular contractions, which may be unsuitable for people suffering from joint disorders. The neuromuscular electrical stimulation (NMES) method which is increasingly used by physiotherapists (de Oliveira Melo et al. 2013; Papadopoulos et al. 2013) enables to alleviate these aforementioned constraints and can be performed at home, provided specific cautionary advice is given (Quittan et al. 2001).

Depending on the characteristics of the training program, NMES has identical or even greater benefits compared to voluntary muscle training (Maffiuletti 2010). For example, as pointed out by the author, when compared to voluntary training and conventional rehabilitation procedures, NMES (regardless of whether it is combined with voluntary exercise) is more effective in preserving muscle function during a phase of reduced activity/immobilization (Bax et al. 2005; Vivodtzev et al. 2006; Glinsky et al. 2007) and is equally effective in recovering muscle function after an immobilization period (Bax et al. 2005). Although the muscle structure and functional gains generated by NMES are largely described in healthy individuals and some pathological conditions, such as cardiac disease or people with chronic obstructive pulmonary disorder (for a review, see Maffiuletti 2010), only a few studies have investigated this technique in older people (Caggiano et al. 1994; Amiridis et al. 2005; Paillard et al. 2005; Kern et al. 2014). Furthermore, the effect of NMES on the muscle-tendon properties remains poorly documented in all populations. A recent study by Gorgey and Khalil (2015) has shown preliminary results on a small group comprising four spinal cord injured individuals, who were trained with a 30 Hz NMES protocol on knee extensor muscles over 12 weeks. The training leads to a nonsignificant increase in cross-sectional area of the patellar tendon (+8%, *P* = 0.14). Another study by Grosset et al. (2014) has shown that 4 weeks of high-frequency NMES training in TS muscle of young sedentary participants induced positive changes in the contractile and elastic properties of the muscle-tendon complex. They hypothesized that a fiber-type transition to fast fibers is a more likely scenario compared to slow fibers without any change in passive (i.e., tendon) stiffness. Nevertheless, fiber-type transition toward faster fiber type after NMES still requires clarification as some results were in agreement with those previous findings (Perez et al. 2002; Kern et al. 2014) whereas others showed a fiber-type transition toward slower fibers (Maffiuletti et al. 2006; Gondin et al. 2011). Finally, Grosset et al. (2014) suggested that high-frequency NMES training could lead to functional changes of particular interest in older adults. It is known that the stiffness of the muscle-tendon complex involved in the transmission of forces between the skeleton and muscles is related to the activation of proprioceptive organs during sudden stretching or significant postural sway (Woollacott et al. 1986). This point is in line with the onset latency of the postural TS muscle noted in response to a body sway in older adults when compared to younger adults, suggesting a longer proprioceptive processing time (Amiridis et al. 2005).

Most of the time, the NMES training is passive without voluntary participation. Contrary to voluntary muscle training where postural balance control is required, the NMES training is widely conducted in a sitting or lying position with very low involvement of postural control. However, it is still unclear whether the NMES-trained participants can take full advantage of expected improvements in muscle-tendon properties during ecological situations where the postural balance is engaged.

### Study aims and experimental setup

The present study targeted pre-frail people recently admitted to a long-term care facility. Due to their environmental changes and the related full daily life assistance, this population is of particular interest, as the transitional period potentially leads to a loss of autonomy, which is a hallmark of impairment in motor-balance control.

This study aimed to (1) evaluate the potential MT changes and the associated postural control improvement induced by NMES training in pre-frail older adults, (2) evaluate the ability of NMES-trained participants to transfer the force and force transmission gains into functional performance through a challenging postural task and finally, (3) determine whether these systemic changes affect the postural sway in a demanding low-force standing task.

Two main experimental setups were designed to meet this aim. First, the force level and force transmission characteristics of the trained muscle were assessed during maximal voluntary contraction (MVC) of the subject in lying position. This measure was achieved with an ergometer synchronized to an ultrasonography device. Second, in standing position, the dynamic behavior of CoP was assessed during two different tasks: bipedal quiet standing and LoS. For all measurements, the performance of the NMES-trained group was compared before and after the training period, with a reference nontrained group comprising non-frail older adults living at home.

## Methods

### Population

Sixteen older women took part in the study. Nine of them who resided in a long-term care facility (mean age 82.2 ± 4.4 years old, mean body size 1.56 ± 0.1 m, mean body weight 66.7 ± 12.5 kg) followed the NMES training (NMES group). The reference group (i.e., without NMES training) comprised seven older women (mean age 74 ± 4.6 years old, mean body size 1.6 ± 0.07 m, body weight 67.7 ± 6.1 kg) who were living at home and performed only the pre- and post-session tests.

None of the participants had previous experience with the NMES training protocol, experienced a fall, or exhibited significant cardiac, neurological, cognitive or musculo-skeletal impairments. All of them were fully autonomous in their daily activities, as evidenced by the Katz score: 6/6 for all enrolled participants (Katz et al. 1970). All participants reported normal or corrected to normal vision (e.g., glasses, contact lenses).

In the framework of this study, participants in the NMES (institutionalized) group were considered as pre-frail older adults based on their autonomy level, and they showed a strong reduction in the execution speed of functional tasks in comparison to the reference group (Timed Up and Go test performance = 5.3 ± 1.1 sec vs. 12.6 ± 6.7 sec for NMES group and control group, respectively, *u*(14) = 3.33, *P* = 0.0009). Since the reference group comprised younger individuals with a superior functional status (significantly higher score at the Timed Up and Go test) and a different lifestyle (living at home) compared to the NMES group, their reported performances were not considered as control values but as reference values in this study. Written informed consent was obtained for each participant, and the studies conformed to the standards set by the latest revision of the Declaration of Helsinki. The procedure was approved by the local geriatric ethics committee of the hospital of Beaune-21200, France.

### Experimental design

**Figure 1:**
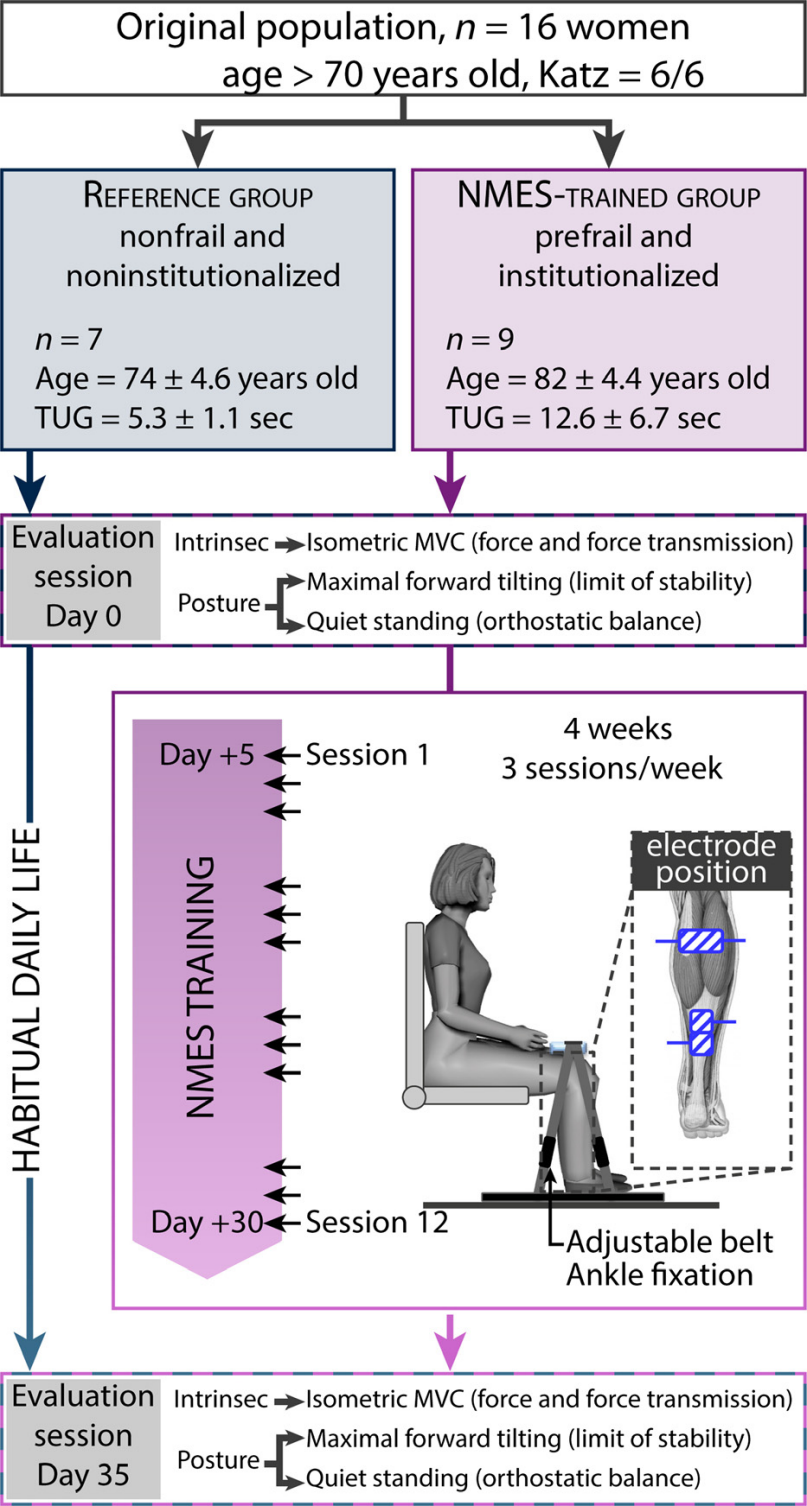
Experimental design. All of the enrolled participants were older than 70 years old and still fully autonomous (maximal score to Katz test), but NMES-trained participants had a lower performance on the functional “Timed-up and Go” test in comparison to their nontrained counterparts who constituted the reference group. Thirty-five days separated the two evaluation sessions. The main objectives of the evaluation sessions were the assessment of some intrinsic muscle and tendon properties during maximal voluntary contractions as well as the postural control characteristics during a challenging and quiet standing task. NMES training comprised two evaluation sessions conducted three times per week for 4 weeks. NMES-trained participants performed the training in sitting position, with the ankle, knee, and hip joints at 90°. The legs remained fixed by an adjustable belt that guaranteed isometric contraction of stimulated muscles.

On day 0, all participants completed the evaluation session (pre-session) (Fig.1), which consisted of a full set of tests, including isometric maximal effort and the assessment of postural sways during LoS and quiet standing. Then, the NMES group underwent the NMES training program involving 12 sessions conducted three times per week for 4 weeks (Fig.1). In the meantime, during this training period, the reference group continued with their habitual life without performing any specific physical training. Overall, 35 days after the pre-session, all participants completed the second, identical evaluation session (post-session).

### Neuromuscular electrical stimulation training

No specific muscle training protocol was performed at least 3 months prior to the inclusion date. Training sessions were conducted in the sitting position to avoid pain or discomfort, and to prevent unexpected muscle fatigue that may result from the adoption of an unusual muscle position. Additionally, the sitting position facilitated interaction with the experimenters. Ankle, knee, and hip joints were fixed at 90° by an adjustable strap that maintains the position of the entire lower limb during the activation of TS muscle (Fig.1). Self-adhesive electrodes were placed on each leg with two cathodes (5 cm × 5 cm) with membrane-depolarizing properties positioned over the superficial aspect of soleus (SOL) muscle about 5 cm in distance from where the two heads of gastrocnemii join the Achilles tendon. The anode (10 cm × 5 cm) was placed along the middorsal line of shank, over both medial and lateral gastrocnemii. As used and described by Gondin et al. (2006), this configuration allowed coverage of the entire TS muscle (Fig.1).

The NMES sessions were performed with the Energy Compex® device, and they consisted of 25 min of proper training with trains of stimulation (4 sec “ON” separated by 12 sec of rest, rectangular-wave pulsed currents lasting 350 *μ*sec delivered at 100 Hz) that generated a hundred isometric contractions of the targeted muscle. For each training session and each participant, the starting stimulation intensity was set as the lowest intensity eliciting movement at the ankle joint, which was visually detected by a clear elevation of the knee when the participants were in the sitting position. Then the aforementioned adjustable strap was attached to the participant and the training started with a stimulation intensity which was reassessed every 2 min to reach the maximum tolerance threshold without causing any uncomfortable sensation, as described by the physical therapists (see Maffiuletti 2010). The 95% confidence intervals for the full-sample stimulation intensities were between 50 mA and 90 mA. This large range is explained by the interindividual variability in sensitivity thresholds. There was 100% compliance by each participant from the NMES group during the entire training session.

### Torque, EMG assessments, and related data processing

**Figure 2:**
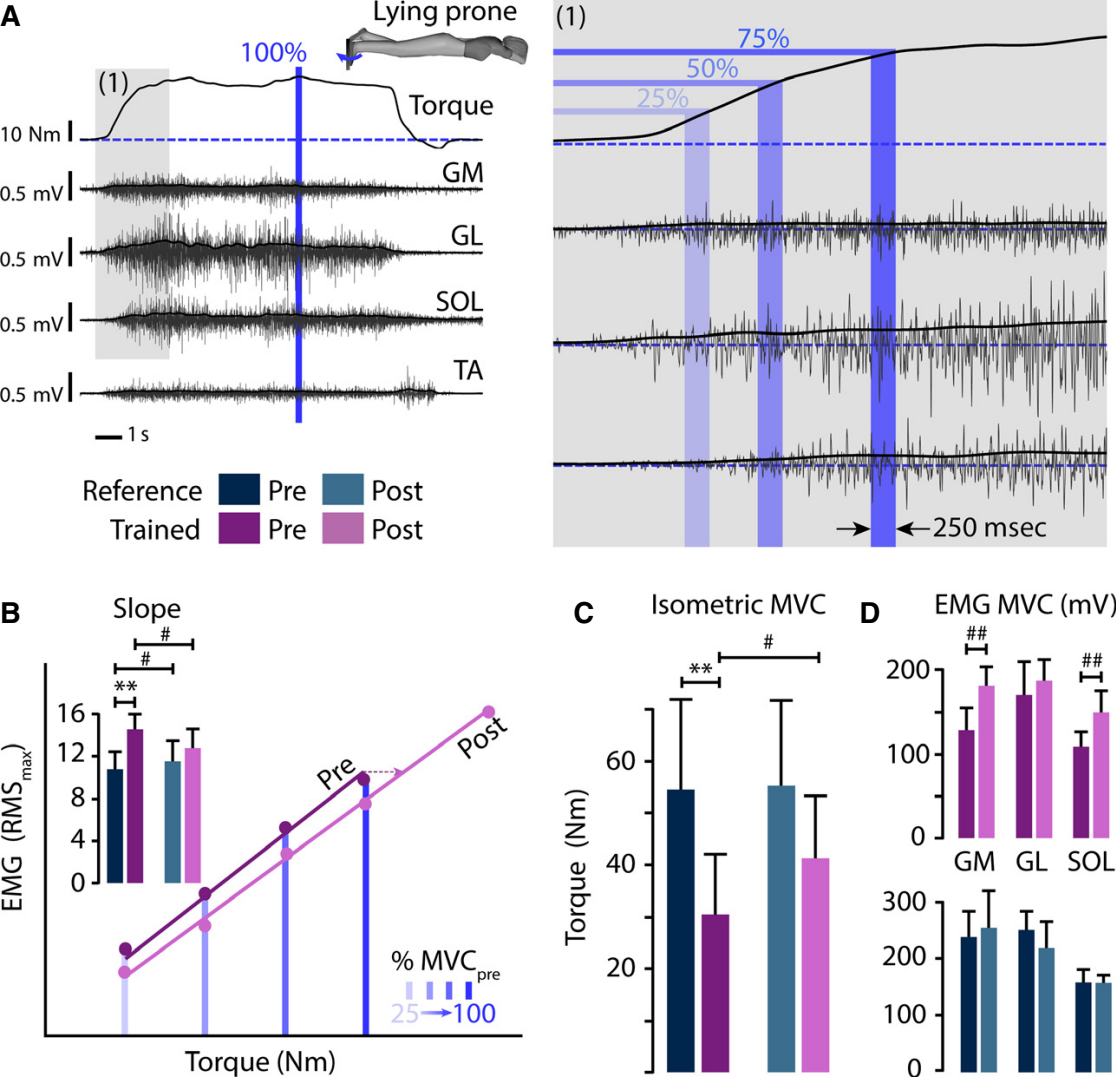
Method overview for the extraction of studied neuromuscular outcomes. Participants were lying prone and performed a progressive maximal voluntary contraction (MVC) in isometric plantarflexion (MVC marked by the blue “100%” line). (A) EMG activities of triceps surae (TS) muscles (GM, GL, and SOL) were recorded together with the main antagonist muscle (TA) and the net torque at ankle joint. (B) Relation between EMG activities of TS muscles and torque developed during plantar flexion. EMG activities were obtained from the sum of the RMS of each three TS muscles during 250 msec around the time points corresponding to 25%, 50%, 75%, and 100% of MVC_pre_ during the ascendant part of the contraction (1). The EMG / Torque relation is presented for a NMES participant at pre- (purple line) and post- (pink line) evaluation sessions. The plotted histogram shows the overall result in terms of the mean slope for both groups at both sessions. (C, D) Histograms of MVC in isometric plantarflexion (C) and root mean square (RMS) of EMG activities of the three TS muscles (D). #*P* < 0.05; ## and **P < 0.01.

Similar to Duclay et al. (2009), the participants laid prone on a test bench with their knee joints at full extension and ankle joints at 90° (Fig.2A). Measurements were made on the right foot, which was attached to the footplate of an ergometer (model OMF06M, OMICRO’N, Gambais, France). The rotation axis of the device was aligned with the anatomical ankle flexion–extension axis. After 2 min of muscular warm-up, the participants were instructed to gradually increasing their force from resting state to MVC within 4 sec to reach a plateau of maximal torque (Fig.2A). A visual feedback of the developed torque was displayed in real time to help the participants appreciate their performance and to motivate them to deliver a true maximal effort (Fig.2A). The task was repeated four times per participant, with 1 min rest between each attempt. The best attempt (i.e., the one showing the highest MVC) was selected for the subsequent data processing (Fig.2B).

To assess the contribution of neural versus peripheral factors during potential muscle force gain (Moritani and de Vries 1979), EMG activity was also collected during the gradually increasing force task. EMG activities from the TS were recorded by means of 10-mm diameter silver-chloride surface electrode, with an interelectrode distance of 25 mm. The pair of electrodes for the SOL muscle was placed on the middorsal line of the leg, 5 cm below the MT junction of TS. For the gastrocnemius medialis (GM) and gastrocnemius lateralis (GL) muscles, the electrodes were fixed lengthwise over the middle of the muscle belly, following the European Recommendations for Surface Electromyography. The EMG activity of the main plantarflexor antagonist muscle, that is, the tibialis anterior (TA) muscle, was recorded during both PF efforts and dorsiflexion efforts (MVC tasks) in order to visualize and compute the coactivation level. The TA EMG electrodes were placed on the line at 1/3 distance between the tip of the fibula and the tip of the medial malleolus.

Torques and EMG signals were concurrently acquired at a sampling frequency of 5 kHz and processed with a multichannel analogue–digital converter (Biopac Systems Inc., Goleta, CA). TS EMG activities during PF were quantified using the root mean square (RMS) of the processed EMG signals of SOL, GM, and GL independently (Fig.2D) and together (sum, Fig.2B). The RMS values were calculated over a 250 msec period (i.e., 125 msec before and 125 msec after a defined time) at different time points during the gradually increasing force task, at pre-session maximal torque (100% MVC_pre_): 25% MVC_pre,_ 50% MVC_pre_, 75% MVC_pre_, and post-session maximal torque (100% MVC_post_).

For each of these time points, the RMS was plotted for the entire TS as a function of the related torque, and a linear regression was computed (Fig.2B). The change in the slope properties of the linear regression offered a way to discriminate between contributions from neural (upstream to neuromuscular junction) versus peripheral factors (downstream to neuromuscular junction) in order to reveal the potential mechanisms underlying the force gain change following the training period (Moritani and de Vries 1979).

### Musculo-tendinous behavior and related data processing

**Figure 3:**
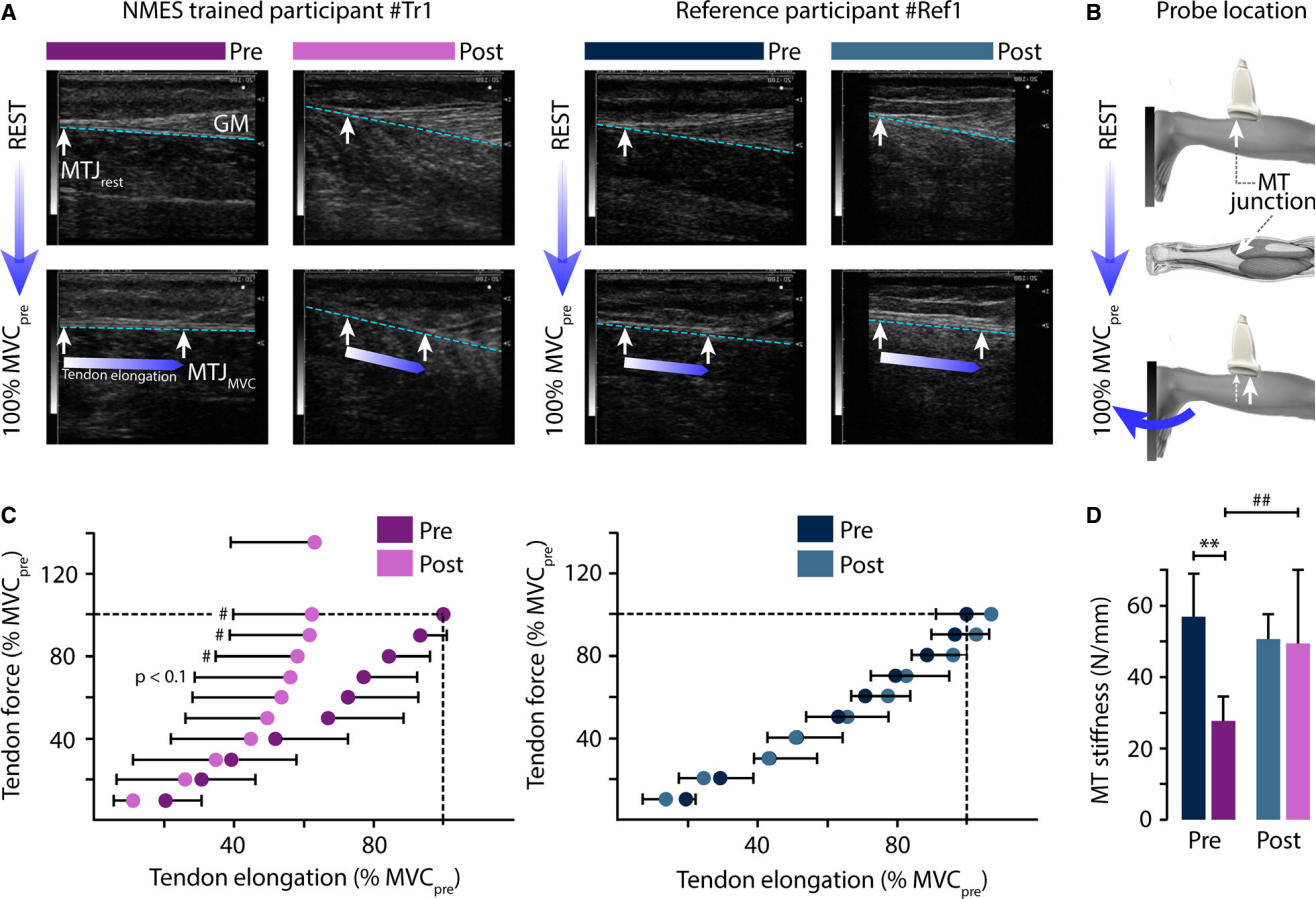
Evolution of musculotendinous junction properties. (A) Ultrasound images of musculotendinous (MT) junction elongation of the TS muscle during maximal voluntary contraction (MVC) in isometric plantarflexion for one participant of each group at both evaluation sessions. During MVC, the displacement of MT junction (white arrow) moves proximally, following the separation line between GM and SOL muscles (blue dashed line) (A, B). For the reference participant, the MT junction elongation for the same effort was not reduced at the post-session (white-blue arrow), and it clearly declined for the NMES-trained participant (A). (C) The mean relationship of tendon force/tendon elongation for each group at both evaluation sessions. The two relations are overlapping for the reference group. A reduction in elongation appears for high force values in the NMES-trained group. (D) Histogram of MT stiffness showing a strong increase for the trained group following NMES training, reducing the initial difference. #*P* < 0.05; ** and ##*P* < 0.01.

During the aforementioned force task, the displacement of the MT junction was assessed with a 7.5 MHz linear-array B-mode probe (Esaote Biomedica®, AU5, Firenze, Italy). As described by Duclay et al. (2009), MT junction displacement was measured by tracking the displacement of GM during gradual isometric PF contraction (Fig.3A and B). Tendon elongation was determined as the distance covered by the MT junction from rest to MVC relative to an external marker placed on skin (Fig.3A and B). Tendon elongation was assessed for different torques, ranging from 10% to 100% MVC during the pre-session (MVC_pre_) with an increment of 10%, and one supplementary measure was included during the post-session (100% MVC_post_).

Tendon force was calculated by dividing the externally measured moment by the moment arm of tendon (set at 0.05 m, which corresponds to the mean value of Achilles tendon moment arm for an ankle joint fixed at 90°). MT stiffness was calculated at high contraction (at 100% MVC_pre_) by dividing tendon force by tendon elongation. The average relationships between tendon elongation and tendon force are presented in Figure3C and D.

### LoS assessment and related data processing

**Figure 4:**
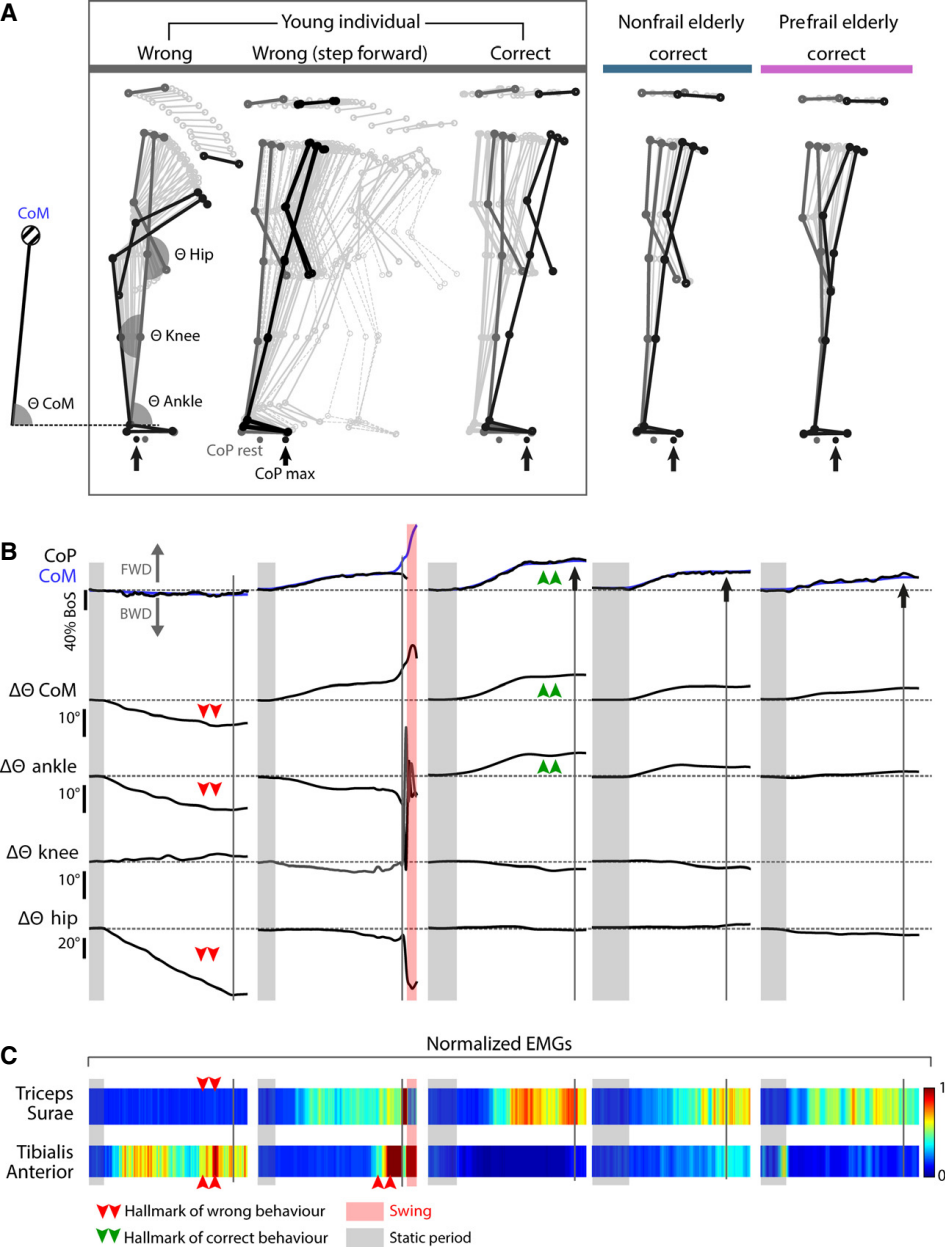
Kinematic (A, B), kinetic (B), and electromyographic (C) illustration of maximal limit of stability (LoS) task performed by a young individual and two elderly participants (non-frail and pre-frail). Three trials are presented for the young individual (grey panel) to better identify the hallmarks of a wrong versus a correct behavior. The trial on the left shows a hip flexion leading to a backward displacement of the center of mass (CoM) (i.e., opposite to expected direction) and absence of activation of targeted muscles (TS muscles). In the second trial, the subject did a step at the end of the attempt because CoM position has exceeded the LoS. The last trial of the young individual displays the correct behavior: CoM and center of pressure (CoP) are moving forward, with an isolated motion of ankle angle (hip and knee joint angle remain fixed) and an activation of the targeted muscles without significant co-contraction (silent TA). The two trials on the right show that both non-frail and pre-frail elderly participants were able to perform the task by respecting the afore-described kinematic, kinetic, and EMG hallmarks of the correct behavior.

As defined by Melzer et al. (2009), LoS can be described as the maximal A-P distance a person can intentionally displace the projection of his CoM within the BoS by leaning his body in a given direction without losing balance, stepping, or grasping. The participants were asked to lean as much as possible toward the anterior BoS boundary by tilting their entire body in the forward direction while keeping their arms along the trunk and their hip joints open without moving their feet. Two experimenters validated the participants’ behavior during the habituation trials. The capacity of the participants to correctly perform the task was tested through kinematic, kinetic, and EMG evidence obtained from representative participants from both studied group (Fig.4). Inappropriate movement (no forward movement of CoM, strong hip flexion, and no activation of ankle extensor muscles, or when making a step forward) performed by a young non-frail individual during the first spontaneous trial was corrected through demonstration in Figure4. This latter illustrates that older adults from both studied groups were fully capable of properly performing the LoS task.

Each trial started with 5 ± 2 sec of quiet standing, followed by 25 ± 5 sec with two to four maximal LoS attempts. No strict rhythmic tilting was imposed on the participants in order to record their spontaneous dynamic postural behavior. Three valid trials (correct kinematic behavior and without moving feet) per participant were selected for the data analysis.

**Figure 5:**
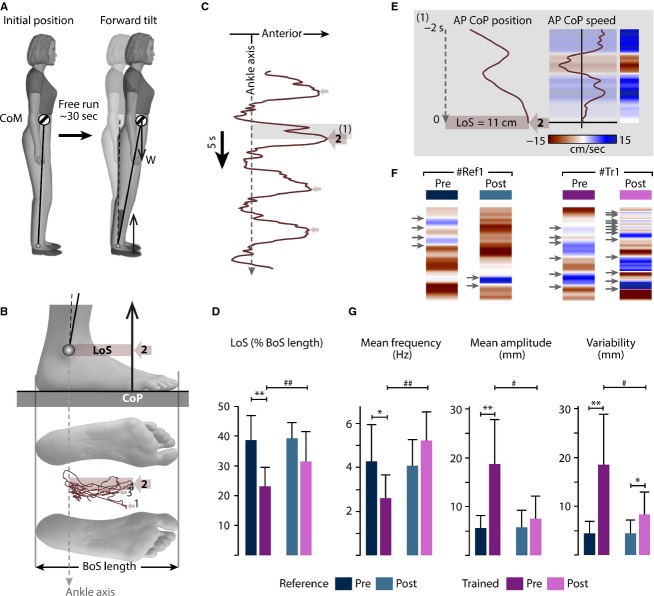
Limit of stability (LoS) task and data processing. (A) Illustration of the task from initial position to maximal forward tilt. From a bipedal standing position, participants were asked to reach and stay at the LoS in forward direction during approximately 30 sec. (B) Trajectory of the center of pressure (CoP) within the base of support during the task. For the presented trial, the maximal forward position of CoP was reached at the second sway (#2). (C) Anteroposterior (AP) position of CoP overtime. (D, G) Histogram representation of the extracted variables for each group and for both evaluation sessions: LoS (D) and the others computed CoP parameters 2 sec prior to reaching the LoS (G). (E) Analysis of AP CoP speed focused on 2 sec prior revealed the instantaneous speed and CoP sway frequency (color changes from blue to brown and inversely), with arrows indicating changes in the direction of postural sways. (F) Color code representation of the AP speed of CoP during the 2 sec time window prior to the LoS for one representative participant of each group. While the overall behavior seemed unchanged for the reference participant, a clear increase in maximal speed and frequency emerged for the NMES-trained participant. * and #*P* < 0.05; ** and ##*P* < 0.01.

The CoP trajectories were recorded using a force platform. The maximal forward position of CoP (CoP_max_) for each trial was used to calculate the forward LoS, that is, the A-P distance from ankle axis to CoP_max_ (Fig.5A–C). To compare individuals with different height and feet sizes, LoS has been expressed as the percentage of BoS length, that is, the distance between anterior- and posterior boundaries (King et al. 1994; Schieppati et al. 1994).

To quantify the behavior of postural sways closed to CoP_max_, the A-P speed of CoP has been calculated by deriving the A-P CoP position 2 sec prior and 2 sec after CoP_max_. Representation of CoP speed through a color code enables easy visualization of the sway behavior (Fig.5E and F). Mean frequency, mean amplitude, and amplitude variability were calculated from two time windows over the 2 sec prior to the CoP_max_ and the 2 sec after CoP_max._ Moreover, this 2 + 2 sec corresponded to the observed minimal time to complete the full backward and forward movement of the tilting task among all recorded attempts.

The torque generated at the right ankle when reaching CoP_max_ was computed by integrating the vertical offset from the forceplate level and the ankle joint level. Then, the index of used force (IUF), that is, the index of force developed during standing compared to the intrinsic performance, was calculated as the ratio of torque at CoP_max_ and MVC during prone position for the corresponding evaluation session.

### Quiet standing assessment and related data processing

Participants were asked to stand quietly and barefoot with eyes open. They were instructed to look straight ahead with the arms alongside their body and stare at a visual reference mark placed at a 2 m distance in front of them. Each foot was positioned on the force plate (sampling frequency of 40 Hz), such that the distance between the medial side of the heels was 8.4 cm with an external rotation angle of 9°. The postural test consisted of three 30 sec trials with this quiet stance. For both A-P and M-L directions, three CoP variables were considered to describe the orthostatic postural control performance. The maximal range of CoP displacements indicated the maximal excursion of CoP in any direction. The speed of CoP displacements was calculated as the sum of the scalar displacements (i.e., cumulated distance over the period of interest) divided by the sampling time. This measure has been shown to represent a key variable in the maintenance of postural stability, thereby providing essential functional information about the true nature of postural control in older adults (Maki et al. 1990; Deschamps et al. 2013).

### Statistics

Nonparametric tests have been used to compare all the samples for each variable. Mann–Whitney tests were applied for the intergroup comparison (*P < 0.05, **P < 0.01, ***P > 0.001) and Wilcoxon signed-rank tests were applied for the paired samples comparison (^#^*P* < 0.05, ^##^*P* < 0.01, ^###^*P* > 0.001).

#### Principal component analysis

**Figure 6:**
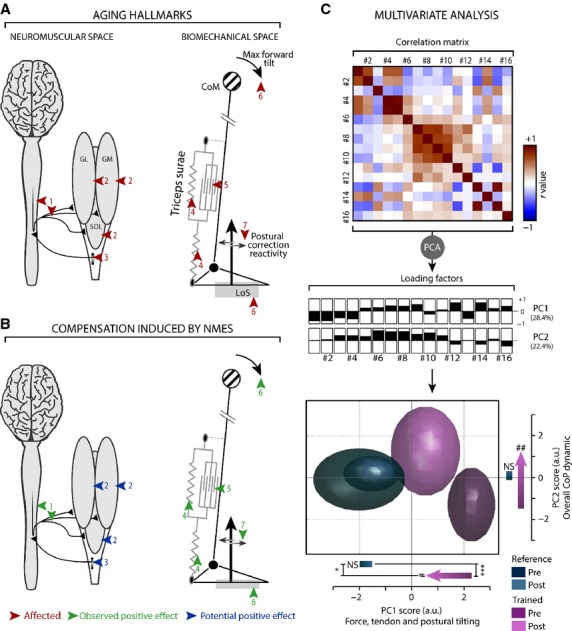
(A, B) Model highlighting the main neuromuscular and biomechanical elements involved in control of posture during forward body tilting, as discussed in the study. On the left side, the targeted muscles (GL, GM, and SOL) are represented with their tendon unit and their connections to the central nervous system through sensory and motor nervous fibers. On the right side, the overall body is sketched as an inverted pendulum with its center of mass (CoM) that oscillates around ankle joint. GL, GM, and SOL muscles are grouped together, forming the triceps surae (TS) complex, which is modeled by a contractile element and an elastic component in parallel while TS tendon is represented by a series of elastic components linked to the foot. Red arrows indicate the different sites affected by physiological aging: (1) neural descending pathway, including motoneurons, (2) muscle structures of TS muscle, (3) sensory pathway from Golgi Tendon Organ, (4) elastic components of muscle-tendon unit, (5) contractile component of the TS muscle, (6) maximal limit of stability of CoM and the related projected area within the base of support (LoS), and (7) dynamic reactivity to move center of pressure to correct postural tilting. Green arrows point to the elements that improved after the NMES training from the analyzed statistical results. Blue arrows show the potentially improved elements, which have to be corroborated with the quantified results. (C) Multivariate analysis was performed on the collected variables, including all of the studied components, to provide an overall vision of the evolution of each studied group. The correlation matrix of the 16 raw variables to be used in principal component analysis is presented. The histogram of loading factors represents the correlation of each variable with the corresponding principal component (PC). The two first PCs together explain 50.8% of the total variance. On the bottom part of panel C, the overall results of PCA in the 2-D space are defined by the two first PCs. Performances of all participants are clustered in ellipsoid surfaces according to their group and evaluation session. While for reference group, pre and post surfaces are overlapping, the surface of the NMES group significantly moved toward the area of the reference group, which provides visual and quantified evidence that NMES training reduces the difference between the pre-frail group and the reference group. * and #*P* < 0.05; ##*P* < 0.01; ***P < 0.001.

To offer an overall vision of the evolution of pre-frail group in response to the NMES training and in comparison to reference group, we implemented a multistep statistical procedure based on principal component analysis (PCA). We applied the methodology described in several studies, both in animal and human beings, which used PCA on a database comprising various biomechanical and neurophysiological variables (van den Brand et al. 2012; Mignardot et al. 2014). Briefly, PCA allowed the extraction of the most relevant information from the initial data by generating new, independent variables called principal components (PC). Each PC is a linear combination of the original variables that maximizes the amount of explained variance for each successive PC. The loading factors refer to the computed correlations between each selected PC and each variable. The performances (PC score, Fig.6C) of each participant were clustered according to her group and to the evaluation session at the ellipsoid surface in a 2-D space constructed of the first two PCs. Thus, the above-described statistical analyses were applied to both selected PCs.

The performance of all participants at both time points and for each of the 16 variables used for the PCA has been plotted in scatter plots in Figure S1.

## Results

### Torque and EMG performances in prone position

The analysis of MVC revealed a difference between groups at pre-session (*P* = 0.005), which was mitigated at post-session (*P* = 0.21). In comparison to the reference group that showed no change in performance (*P* = 0.47), the pre-frail group demonstrated an improvement in MVC after NMES training (30.5 ± 12.5 vs. 41.3 ± 12.8 Nm, i.e. +26.2%, *P* = 0.039, Fig.2C, Tables2). This result can be linked to their stronger EMG activity of their GM and SOL muscles (Fig.2D, Tables2) and the reduction in the slope of the linear regression between EMG activity and torque (14.6 ± 1.4 vs. 12.8 ± 1.8, *P* = 0.027, Fig.2B, Tables2).

**Table 1:** Mean value with standard deviation of all the variables used for the Principal Component Analysis and their outcomes in terms of loading factors on PC1 and PC2 for both groups and both time points

Variables	Number	NMES trained		Reference		Loading factors (PCA)	
		Pre	Post	Pre	Post	PC1	PC2
Neuromuscular performance (lying)							
Isometric MVC (Nm)	1	30.5 ± 12.4	41.3 ± 12.8	54.6 ± 18.9	55.2 ± 17.8	−0.80	0.18
Regression slope – Torque versus EMG	2	14.6 ± 1.4	12.8 ± 1.8	10.8 ± 1.7	11.5 ± 1.9	−0.01	−0.44
MT stiffness index (N mm^−1^)	3	27.8 ± 7.3	49.5 ± 21.9	56.9 ± 21.7	50.9 ± 20.2	−0.75	0.25
Limit of stability task (standing)							
Limit of stability, LoS (% BoS length)	4	23.1 ± 6.5	31.5 ± 10.0	38.6 ± 8.3	39.1 ± 5.4	−0.57	0.51
Torque at LoS (Nm)	5	19.0 ± 8.2	24.3 ± 11.3	32.7 ± 10.0	32.7 ± 6.4	−0.49	0.51
Index of used force	6	0.7 ± 0.3	0.6 ± 0.2	0.6 ± 0.3	0.6 ± 0.2	0.35	0.28
CoP characteristics (2 sec prior LoS)							
Frequency (Hz)	7	2.6 ± 1.1	5.2 ± 1.3	4.3 ± 1.7	4.1 ± 1.2	−0.16	0.67
Amplitude (mm)	8	18.8 ± 9.1	7.5 ± 4.7	5.6 ± 2.4	5.7 ± 3.5	0.63	−0.42
Variability (mm)	9	18.9 ± 10.3	8.6 ± 4.6	4.8 ± 2.5	4.7 ± 2.7	0.65	−0.43
CoP characteristics (2 sec after LoS)							
Frequency (Hz)	10	3.7 ± 1.0	5.4 ± 2.2	3.7 ± 1.5	4.1 ± 1.1	0.20	0.39
Amplitude (mm)	11	18.2 ± 5.7	15.5 ± 8.0	37.1 ± 23.2	28.8 ± 14.0	−0.58	0.13
Variability (mm)	12	14.3 ± 4.5	16.5 ± 9.1	12.6 ± 10.2	12.8 ± 15.8	0.26	0.13
Quiet standing balance (CoP sway)							
Mean speed M-L (mm s^−1^)	13	5.8 ± 2.8	9.0 ± 3.5	5.4 ± 2.7	6.8 ± 1.7	0.44	0.74
Mean speed A-P (mm s^−1^)	14	13.2 ± 5.7	16.5 ± 6.9	10.4 ± 3.6	9.8 ± 2.5	0.57	0.65
Max range M-L (mm)	15	16.8 ± 7.6	22.0 ± 10.0	11.6 ± 3.1	16.3 ± 5.5	0.55	0.67
Max range A-P (mm)	16	28.0 ± 9.0	25.2 ± 8.7	18.7 ± 5.9	18.4 ± 4.7	0.65	0.47
Principal component analysis							
Score on PC1 (a.u.)		2.3 ± 0.9	0.5 ± 1.35	−2.0 ± 1.8	−1.5 ± 1.1		
Score on PC2 (a.u.)		−1.3 ± 1.8	1.3 ± 2.15	−0.1 ± 1.5	0.2 ± 0.9		

**Table 2:** Inter-group (Mann-Whitney, U-Test) and intra-group (Wilcoxon signed rank, WSR-Test) statistical outcomes

Variables	Number	INTER-group				INTRA-group			
		Pre		Post		Reference		NMES	
		*U*	*P*	*U*	*P*	WSR	*P*	WSR	*P*
Neuromuscular performance (lying)									
Isometric MVC (Nm)	1	6.0	**0.005**	19.0	0.21	9.0	0.469	3.0	**0.039**
Regression slope – Torque versus EMG	2	61.0	**0.001**	44.0	0.199	0.0	**0.016**	41.0	**0.027**
MT stiffness index (N mm^−1^)	3	5.0	**0.003**	32.0	0.978	19.0	0.469	1.0	**0.008**
Limit of stability task (standing)									
Limit of stability (% BoS length)	4	3.0	**0.001**	13.0	0.051	10.0	1.000	1.0	**0.008**
Torque at CoPmax (Nm)	5	9.0	**0.016**	17.0	0.142	11.0	1.000	4.0	**0.027**
Index used force	6	35.5	0.722	30.5	0.997	16.0	0.813	32.0	0.301
CoP characteristics (2 sec prior LoS)									
Frequency (Hz)	7	13.0	**0.050**	49.0	0.066	16.5	0.730	0.0	**0.009**
Amplitude (mm)	8	60.0	**0.001**	41.5	0.315	14.0	0.938	42.0	**0.020**
Variability (mm)	9	61.0	**0.001**	51.0	**0.039**	14.0	0.938	41.0	**0.027**
CoP characteristics (2 sec after LoS)									
Frequency (Hz)	10	34.5	0.775	44.5	0.18	5.5	0.684	8.0	0.095
Amplitude (mm)	11	16.0	0.108	14.0	0.071	24.0	0.108	29.0	0.496
Variability (mm)	12	33.0	0.892	44.0	0.21	16.0	0.800	21.0	0.910
Quiet standing balance (CoP sway)									
Mean speed M-L (mm s^−1^)	13	39.5	0.426	44.5	0.181	6.0	0.205	1.0	**0.008**
Mean speed A-P (mm s^−1^)	14	39.5	0.425	53.0	**0.021**	15.0	0.933	6.0	0.055
Max range M-L (mm)	15	48.0	0.091	42.5	0.264	0.0	**0.022**	7.0	0.074
Max range A-P (mm)	16	50.0	0.055	43.5	0.221	14.0	1.000	31.0	0.359
Principal component analysis									
Score on PC1 (a.u.)		63.0	**0.0002**	56.0	**0.011**	6.0	0.205	42.0	**0.024**
Score on PC2 (a.u.)		19.0	0.204	40.0	0.397	10.0	0.554	0.0	**0.009**

Bold values are statistical significance threshold crossed.

### Musculo-tendinous behavior

While no significant change in tendon elongation was observed for low and intermediate force values (up to 60% of MVC_pre_), the NMES group revealed a reduction in distance traveled by the MT junction for high force value (*P* < 0.1 for 70% MVC_pre_, *P* < 0.05 for 80% MVC_pre_; Fig.3C). No change was reported for the reference group, regardless of the developed force. The pre-frail group showed the lowest MT stiffness compared to the reference group at pre-session (*P* = 0.003) but they demonstrated significant improvement for this variable/parameter after training (27.8 ± 7.3 vs. 49.5 ± 21.9 N mm^−1^, i.e., +44%, *P* = 0.008, Fig.3D, Tables2), such that no more difference existed between groups at post-session (*P* = 0.978). MT stiffness was computed at 100% MVC_pre_.

### LoS and torque performance during tilting in standing position

While participants from the reference group were able to amply move their CoP when performing the LoS task (38.6 ± 8.3% of BoS length at pre-session), the performance was significantly lower for NMES participants at the pre-session (23.1 ± 6.5%, *P* = 0.001, Fig.5D, Tables2). The statistical analysis revealed a significant improvement in NMES group performance after training (31.5% of BoS length at post-session, *P* = 0.008), with a value that did not differ significantly from that of post-session reference group (*P* = 0.051, Fig.5D, Tables2).

Concerning the torque generated when reaching the LoS, the profile of the results can be regarded as close to those described for the LoS performance (Tables2). The IUF remained unchanged across the groups and the evaluation sessions, with stable values were around 0.65 (Tables2).

### Postural sway during the tilting task

Although no significant change in CoP sway characteristics was observed for the 2 sec after CoP_max_, all calculated characteristics were modified for the NMES group during the 2-sec period prior to CoP_max_ (Fig.5G, Tables2). While the sway frequency for the reference group remained unchanged between sessions (4.3 ± 1.7 Hz at pre-session; 4.1 ± 1.2 Hz at post-session), this variable strongly increased for the NMES group (2.6 ± 1.1 Hz at pre-session; 5.2 ± 1.3 Hz at post-session; *P* = 0.009, Fig.5G, Tables2). The statistical analysis also revealed a decrease in the amplitude (*P* = 0.02) and the variability (*P* = 0.027) of the sway after NMES training, while these characteristics remained stable for the reference group (Fig.5G, Tables2). These outcomes were also transcribed through a color-coded visualization of CoP speed as represented in Figure5F that displays the attempts of two representative participants from both groups.

### Postural sway during quiet standing

No significant change in CoP sway characteristics was observed after the training period regarding the anteroposterior component. Nevertheless, we can note an effect of the session on the mediolateral component of the mean speed for the NMES group (*P* = 0.008, Tables2).

### Principal component analysis

The first two PCs explained the 28.4% and 22.4% of total variance, respectively, and comprised mainly variables related to force, tendon, and postural tilting (PC1) as well as the overall dynamic properties of CoP (PC2). The statistical analysis performed on the score of both plotted PC showed for PC1, while the location of the reference group remained unchanged from pre- to post-sessions (*P* = 0.205), the NMES group differed significantly from the reference group at pre-session (*P* = 0.0002) and became significantly closer to it after the training (*P* = 0.024; horizontal axis on the 2D plot on Fig.6C). Regarding PC2 (vertical axis on the 2D plot on Fig.6C), the statistical analysis also indicated a modification of the location of the NMES group after the training period (*P* = 0.009), while the location of the reference group within the PC-space remained unchanged (*P* = 0.554, Tables2).

## Discussion

The primary aim of this study was to characterize the effects of 4 weeks of TS NMES training on postural control in pre-frail older people. The second aim was to investigate how potential NMES-induced changes in force and force transmission at the level of ankle joint could be associated with changes in postural control, both in challenging LoS and quiet standing tasks. Our results showed that NMES training significantly reduced the differences between pre-frail participants’ and reference participants’ performance on challenging postural control. These results should be seen in relation to intrinsic neuromuscular and musculotendinous gains, which were currently observed in a functional balance context.

### NMES improves voluntary muscle performance through both neural and peripheral adaptations

In agreement with previous results, the maximal voluntary force developed during PF effort was low among older people (Morse et al. 2004; Narici et al., 2004). Although some of the effects of aging in sedentary people on muscle force and its mechanical properties have already been reported (e.g., Ochala et al. 2004), the maximal voluntary force generated at the ankle joint has not been yet documented for older adults based on their residence type and frailty status. First, our results demonstrated that before NMES training, MVC performance while lying was significantly lower for pre-frail institutionalized participants in comparison to healthy noninstitutionalized participants (−44.2%). However, 4 weeks of NMES training significantly improved the performance of trained participants to 26.2% (Fig.2C). Gondin et al. (2005, 2006) identified the mechanisms underlying NMES-related torque increase after 8 weeks training in young adults. Analysis of mid-quadriceps anatomical cross-sectional area demonstrated that an increase in both neuromuscular activation and peripheral adaptation explained the improvement in MVC (Gondin et al. 2005).

The current NMES training was performed over 4 weeks. Regarding this quite short period, we expected that mechanisms underlying the observed gain in torque production would be related mainly to neural adaptation. Accordingly, EMG maximal activity increased for both GM and SOL muscles following NMES training (Fig.2D), reflecting stronger motoneuron activity (Fig.6B).

The relationship between EMG and force on ongoing effort of PF (Fig.2B) confirmed those results. Neural adaptations based on greater EMG activity of the TS muscle and peripheral adaptations, as suggested by reductions in slope (see Fig.2B), explained the force gains. A direct measure of muscle changes through imagery is needed to confirm this hypothesis but NMES seems to have a positive effect against sarcopenia by increasing both the neural descending drive and muscle properties.

### NMES modifies MT properties

Tendon is involved mainly in force transmission from muscles to bones and in fine to external environment, and vice versa. Its mechanical properties determine the speed of force transmission that must be considered when studying postural control. It is already known that the muscle-tendon complex is more compliant with aging (Lexell et al. 1983; Narici and Maganaris 2006; Onambele et al. 2006; Narici et al. 2008), which could be a drawback when posture is suddenly disturbed and the reaction force has to be quickly transmitted to allow rapid postural corrections.

Our results demonstrate that the lower MT stiffness observed in the pre-frail group at the initial stage is reduced after NMES training (+43.9%, Fig.3D) and moved closer to the reference group’s value. As evidenced by echography pictures in Figure3A, the MT junction displacement between rest and MVC_pre_ clearly decreased after NMES training for NMES group, while it remained unchanged for the reference participants. This improvement suggests that short-term NMES training induces a useful decrease in the time required for the force transmission. This point is of special interest when the older individuals need to respond as fast as possible to an unexpected postural perturbation. A stiff tendon of the plantarflexor muscle can quickly transmit the generated force to the BoS in order to adjust the position of CoP below the projection of CoM when the balance needs to be recovered (Morasso et al. 1999; Loram et al. 2005). In addition, the enhanced muscle-tendon force transmission might improve the ability of the central nervous system to finely tune the muscle activation pattern needed to meet the task constraints. This feed-forward control process could be recalibrated based on the sensory information provided by peripheral commands (e.g., Seidler et al. 2004). Thanks to the NMES training, the central nervous system could have become more efficient in predicting the optimal motor response because of optimized feed-forward control and postural control, thus preventing the risk of fall in older adults (Horak 2006; Doumas and Krampe 2010).

To summarize, the improvement in force capacity and force transmission properties through a higher MT stiffness revealed some intrinsic MT and neuromuscular activation changes, as observed with NMES performed in the lying position (without any postural constraints). However, the ability to translate these gains in a functional context where balance is precarious remained to be determined, in particular when performing a task demanding a high force. In this way, we used the LoS task that challenged balance performance.

### The NMES-induced changes in muscle-tendon complex: toward evidence of a positive transfer in postural control

The anatomical joint configuration for this challenging LoS task was close to the studied lying prone position: the knee and hip joints were maintained extended and the ankle joint remained between 85° and 90° (Fig.4B). We evidenced similar kinematic (Fig.4A and B), kinetic (Fig.4B), and electromyographic (Fig.4C) characteristics for one young and two older participants representatives from both studied groups. All participants were fully able to correctly perform the expected behavior (see details in Fig.4). In this respect, a reduced ability in older adults to widely move their CoP toward the BoS boundary (i.e. the LoS) has been reported in many studies (e.g., Cavanaugh et al. 1999; Kozak et al. 2003; Melzer et al. 2009). This deficit was consistently more pronounced for the pre-frail group than the nontrained group (−40.2%, Fig.5D). But the striking result is that the NMES training significantly reduced this difference with an improvement of 26.8% between pre-training versus post-training performance (Fig.5D). The IUF notably allowed to compare the torque generated in standing versus lying prone (intrinsic capacity). For all participants, the IUF remained unchanged around 60–70% of their MVC in the prone position (Tables2, Fig. S1). This finding highlighted the fact that the LoS task was consistent with the reduced level of force for tendon elongation after NMES training (Fig.3C).

Regarding the analysis of CoP sway during the 2-sec time windows prior to CoP_max_ (Fig.5), an interesting evolution in kinematic behavior between pre- and post-session is likely be related to the change in MT stiffness of TS. Indeed, CoP oscillated more frequently and less widely at post-NMES training session. This can be linked to an increased MT stiffness because a stiffer MT complex is de facto more able to faster transmit muscle force variations to the external environment. Inertia can thus be considered as reduced, enhancing both the perception of postural disturbances (sensory pathway) through a higher sensibility of muscle spindles, and the transmission of force to the external environment in order to adjust the posture depending on the perturbation (motor drive). Note that a possible learning effect of the LoS task between the sessions was rejected, since the values from untrained group did not vary.

### Control assessment through quiet standing task

It seemed interesting to test the postural control during a simple task where the developed force is much less important. During quiet standing, the generated force was actually estimated at about 10% MVC in an older population (Billot et al. 2010). In this context, Amiridis et al. (2005) studied the effect of a 4-week NMES training (70 Hz) on TA muscle in older participants. They showed that while the maximal voluntary force during dorsiflexion effort improved, the postural control assessed during bipedal quiet standing task through A-P and M-L CoP range and variability remained unchanged. This is in line with the present study, since the amount (CoP speed) and amplitude (CoP range) of anteroposterior postural sway (directly linked to the torque applied at the ankle joint) remained unchanged after the NMES training (Fig. S1, Tables2). These findings suggest that the current gains are likely to be transposed mainly in postural situations where a significant force is required.

### A PCA supports the positive effect of NMES on the initial deficits

To summarize all of the collected data, we performed a PCA, which simplifies the overall evolution of each group after the 4-week period while maintaining highly precise quantification. The ellipsoid surface, which clustered each individual performance (PCs score) as a function of the group and the session, demonstrated that NMES group significantly moved toward the reference group ellipses after the 4-week period (Fig.6C). The initial variables that contribute to PC1 correlate strongly with each other; they gather components related to force, tendon properties, and performance during the LoS task. Moreover, the NMES group statistically reduced their distance from reference group along the PC1 axis but without totally covering the gap that still separated them (Fig.6C). Concerning PC2, which is mainly related to dynamic behavior of CoP during both standing tasks, the NMES group moved beyond the reference group. This evolution demonstrated that the NMES training effectively influenced the postural control. However, after training, the NMES group exhibited biomechanical behavior that still differed from the reference group. Overall, this original data analysis provides quantified evidence to support the alleviating effect of NMES on broad biomechanical and balance deficits related to physiological aging.

### Methodological considerations

We acknowledge that there are some methodological limitations in our protocol. For example, the knee angle was different during the NMES training and the evaluation protocol. Although the contributions of SOL (monoarticular muscle) and gastrocnemii (biarticular muscles) of TS are knee- or ankle-angle dependent (Cresswell et al. 1995), it is somewhat difficult to precisely determine their contribution in terms of force production during voluntary or electrically induced contraction (Sale et al. 1982; Fouré et al. 2013). Thus, the lying prone position was chosen as the best compromise to avoid some difficulties encountered by the older adults in performing maximal PF without muscular effort for knee extension, as in sitting position. In this way, further investigations are necessary to characterize the force and MT stiffness during the NMES session but also while performing the LoS task. We also concede that MT stiffness assessed through longitudinal ultrasonography imagery does not represent the overall structural muscle and tendon unit. A more complete analysis, including cross-sectional area and muscle architecture, would be useful to better understand the full effect of NMES conditioning on peripheral changes.

## Conclusion

This study provides encouraging evidence that high-frequency NMES training is a useful training paradigm to achieve a positive short-term effect on the muscle-tendon unit impaired by physiological aging. Four weeks of TS NMES training increased both force production and force transmission through changes in MT stiffness. These improvements promoted the postural control under a challenging situation through enhanced capacity to explore the BoS by widely and quickly controlling the center of pressure displacement. Future studies would strengthen these current results by considering various patient populations with a larger sample size to confirm that NMES actively contributes to the fight against neuromuscular impairments and its adverse functional consequences such as falls.

## Supporting Information

**Figure S1.** Scatter plots of each variable used for the principal component analysis for both groups and both time points. All individuals’ data are plotted together with their mean value (diamond) ± the 95% confidence interval.
