# Supplementary figures and images for: Neuromuscular electrical stimulation leads to physiological gains enhancing postural balance in the pre-frail elderly
Source: Physiol Rep. 2015 Jul 30;3(7):e12471. doi: 10.14814/phy2.12471 (PMC4552546; doi:10.14814/phy2.12471)

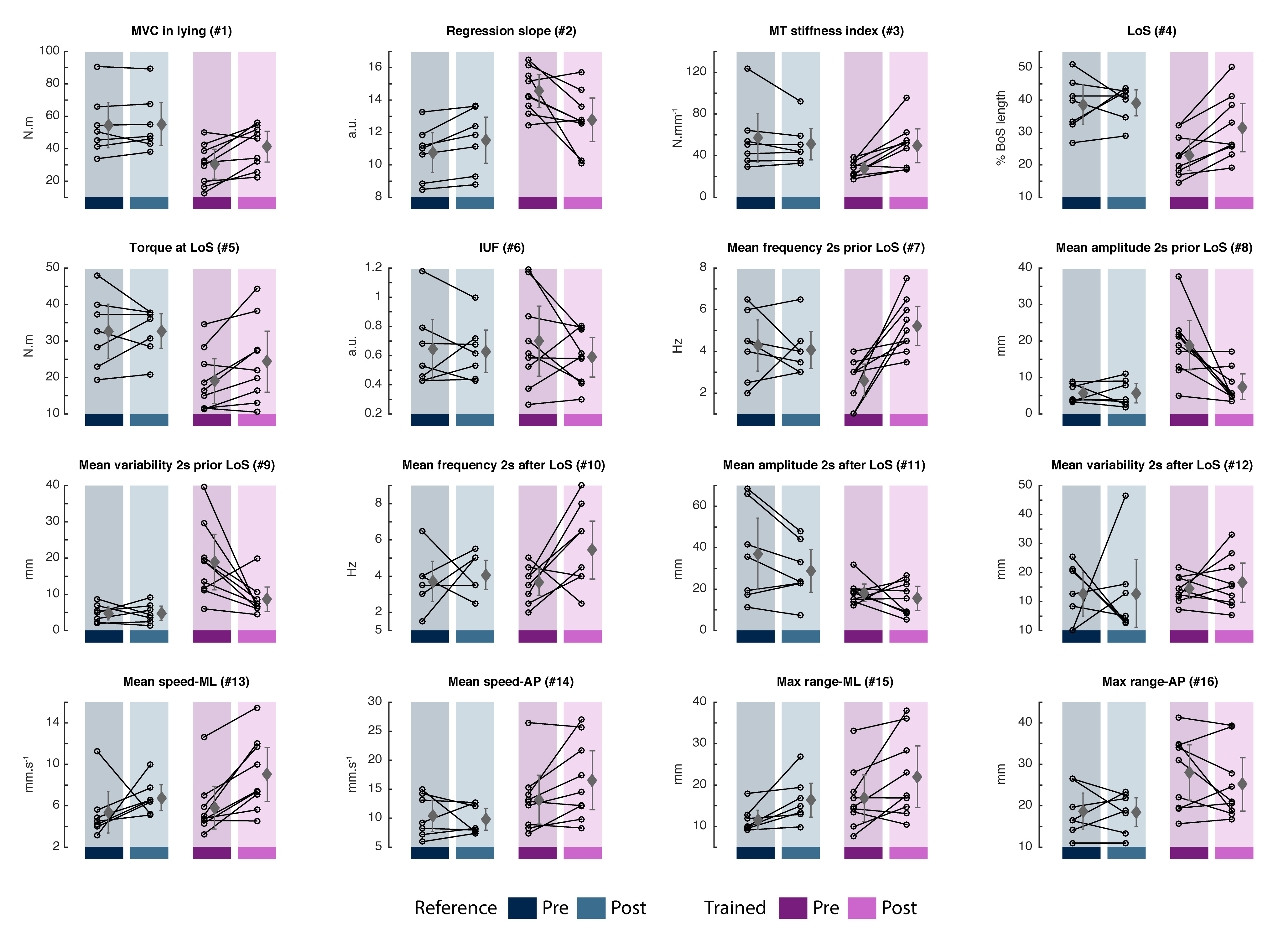

Supplement: Supplementary file 1 [file phy20003-e12471-sd1.jpg]
